# Soluble immune checkpoint proteins as predictive biomarkers for lymph node metastases in penile cancer

**DOI:** 10.3389/fimmu.2026.1754254

**Published:** 2026-02-02

**Authors:** Dominik Glombik, Jessica Carlsson, Peter Kirrander, Sabina Davidsson

**Affiliations:** Department of Urology, Faculty of Medicine and Health, Örebro University, Örebro, Sweden

**Keywords:** liquid biopsy, penile cancer, prediction model, ProcartaPlex immunoassays, soluble immune checkpoint proteins

## Abstract

**Background:**

Penile cancer (PeCa) is a rare but aggressive disease where lymph node metastases (LNM) represent the most significant prognostic factor. Accurate identification of LNM remains a clinical priority, but traditional imaging and clinical parameters often fail to detect occult LNM. Soluble immune checkpoint proteins (sICs) have recently emerged as potential non-invasive biomarkers in various malignancies, although unexplored in PeCa. The primary aim of this study was to explore the value of a panel of 14 sICs for predicting LNM in PeCa. The secondary aim was to compare plasma sIC levels between PeCa patients and cancer-free controls.

**Methods:**

Using ProcartaPlex immunoassays, BTLA, IDO, LAG-3, HVEM, PD-1, PD-L1, PD-L2, TIM-3, CD80, CTLA-4, GITR, CD27, CD28, and CD137 were measured in plasma from 284 PeCa patients and 45 cancer-free controls. PeCa patients were divided into a training set (n=202) and a test set (n=82). A prediction model for LNM was created using logistic regression.

**Results:**

Overall accuracy of the prediction model reached 77.5% (95% CI: 70.9 - 83.3) for the training set, yielding 8.9% sensitivity and 99.3% specificity in predicting LNM. Upon validation using the test set, the accuracy decreased to 62.2% (95% CI: 50.8-72.7) with 17.9% sensitivity and 85.2% specificity. When comparing PeCa patients and cancer-free controls, four inhibitory sICs (IDO, TIM-3, CD80, and CTLA-4) were found at significantly higher levels in the PeCa group. Due to the rarity of the disease, the main limitation of the study is the small number of patients with LNM.

**Conclusion:**

Our study provides no evidence that sICs can predict LNM in PeCa, although four inhibitory sICs were significantly elevated in PeCa patients compared to cancer-free controls, suggesting systemic immunosuppression associated with tumor presence, consistent with findings in other malignancies. Studies with larger cohorts are warranted to clarify the prognostic significance of sICs in PeCa.

## Introduction

Penile cancer (PeCa), predominantly squamous cell carcinoma (SCC), is a rare but aggressive malignancy. The global incidence is approximately 1 per 100.000 men in developed regions, with higher rates reported in parts of South America, Africa, and Asia (1). In Sweden, incidence has increased over the past two decades, now exceeding 2 per 100.000 men (2, 3). The median age at diagnosis is 68 years and established risk factors include human papillomavirus (HPV) infection (4, 5).

PeCa metastasizes primarily to inguinal and subsequently pelvic lymph nodes, and nodal involvement remains the most important prognostic factor (4). A recent Swedish study demonstrated a three-fold increase in disease-specific mortality among patients with lymph node metastases (LNM) (6), while distant metastases (M1) are uniformly fatal (7).

Current imaging lacks sensitivity for detecting occult LNM (8), and no validated predictive biomarkers are available (9–11), hence, clinically node-negative (cN0) patients are stratified into risk-groups based on histopathological features of the primary tumor (12, 13). High-risk patients undergo lymph node staging using dynamic sentinel node biopsy (DSNB), while therapeutic lymph node dissection, due to considerable morbidity, is reserved for those with confirmed metastases (pN+) (14). Hence, an unmet need for sensitive and accessible biomarkers enabling early identification of LNM remains.

Stimulatory and inhibitory immune checkpoint proteins (ICs) are essential regulators of immune homeostasis and play a fundamental role in tumor immune surveillance (15, 16). While benign inflammatory conditions, such as benign prostatic hyperplasia (BPH), may increase circulating ICs, cancer-related immune dysregulation is likely to produce distinct expression patterns or combinations of checkpoints, which may still carry diagnostic or prognostic value. Elevated expression of inhibitory ICs is commonly associated with tumor development, contributing to immune evasion. In contrast, increased expression of stimulatory ICs is more frequently linked to benign inflammatory conditions, reflecting immune activation rather than tumor immune escape. Traditionally, ICs such as PD-1, PD-L1, and CTLA-4, have been studied in their membrane-bound form, mediating cell-to-cell signaling within the tumor microenvironment. Increasing attention has been directed towards their soluble forms (sICs), detectable in plasma and other body fluids (17). These soluble forms retain functional capabilities and can interact with corresponding receptors or ligands at distant sites, potentially modulating systemic anti-tumor immunity.

Several studies have identified sICs as promising biomarkers for both prognosis and prediction of lymph node metastases in various malignancies, including osteosarcoma, pancreatic, kidney, and lung cancers (18–21). However, their role in PeCa remains unexplored, despite documented IC expression in PeCa tissue and growing interest in immunotherapy for advanced disease (11, 15, 22–24). Notably, recent studies have reported high PD-L1 expression, particularly among HPV-positive tumors, supporting further evaluation of immune-related biomarkers in PeCa (22–26).

To date, research on ICs in PeCa has been limited by small cohort sizes and a narrow range of ICs assessed. Larger studies are needed to clarify the biomarker potential of these proteins in PeCa. Given the non-invasive nature and clinical utility of liquid biopsies, evaluating sICs could enhance current diagnostic and prognostic strategies. Ultimately, sICs may emerge as valuable prognostic tools, facilitating earlier detection of LNM, thereby enabling less mutilating treatments and optimally improving survival.

To our knowledge, this is the first study to systematically investigate a broader panel of sICs in PeCa. The primary aim was to evaluate their predictive potential as biomarkers for identifying LNM. Additionally, plasma sIC levels were compared between men with and without PeCa. This study aims to establish a foundation for non-invasive, immune-based risk stratification in PeCa, with potential implications for clinical decision-making in patients with clinically localized disease.

## Materials and methods

### Study population

This cross-sectional study utilized data from the Penile Blood and Urine Study (PenBUS), a Swedish prospective cohort comprising men referred to Örebro University Hospital between 2019 and 2024 for suspected PeCa (N = 316, cases). Men with penile intraepithelial neoplasia (PeIN) and PeCa (pTa-T4) were eligible, while patients with concurrent malignancies, autoimmune disorders, or immunomodulatory therapy were excluded (27, 28). Preoperative blood samples were collected in Lithium heparin tubes and separated plasma was stored at –80°C until further analyses. Patients were stratified into a training set (January 2019 - April 2022) and a test set (May 2022 - July 2024).

A control group included men without PeCa who underwent transurethral resection of the prostate for BPH between 2021 and 2024 (N = 57, controls). The same exclusion criteria and sampling procedures as for cases were applied. Clinicopathological data including age, diagnosis date, tumor characteristics (TNM), and HPV-status among cases were extracted from medical records.

### Soluble immune checkpoint proteins detection

Plasma levels of 14 sICs were quantified using the ProcartaPlex™ Human Immuno-Oncology Checkpoint Panel 1 14 plex (Thermo Fisher Scientific, MA, USA) following the manufacturer´s instructions. Samples from the training set were analyzed on the Luminex 200™ system (Luminex, TX, USA) and those from the test set on the Bio-Plex 200™ system (Bio-Rad, CA, USA).

The panel targets ten inhibitory sICs (B- and T-lymphocyte attenuator (BTLA), Indoleamine 2,3 dioxygenase (IDO), Lymphocyte activation gene 3 (LAG-3), Herpes virus entry mediator (HVEM), Programmed cell death protein 1 (PD-1), Programmed cell death ligand 1 (PD-L1), PD-L2, T-cell immunoglobulin and mucin domain 3 (TIM-3), Cluster of Differentiation (CD) 80, and Cytotoxic T-lymphocyte associated protein 4 (CTLA-4/CD152)) and four stimulatory sICs (Glucocorticoid-induced tumor necrosis factor receptor related protein (GITR), CD27, CD28, and CD137). Data were processed using xPONENT 3.1 (training set) and Bio-Plex manager 6.2 (test set). All samples were analyzed in duplicate and concentrations were calculated from standard curves using a 5-parameter logistic fit.

### Statistical analysis

Samples with concentrations below the lowest standard were set to the lower limit of quantification (LLOQ) and only sICs detectable in >50% of samples were included in further analyses. Data were Box-Cox transformed to approximate normality. Continuous variables were described as means and standard deviations and categorical variables as absolute and relative frequencies. Group differences were compared using Welch´s t-tests or ANOVA for continuous variables and Chi-square or Fisher´s exact tests for categorical variables. One-tailed t-tests were applied when comparing sIC levels between cases and controls, and between LNM-positive and LNM-negative patients; all other tests were two-tailed.

Multiple testing was adjusted using the Benjamini-Hochberg false discovery rate (FDR) procedure. A logistic regression model predicting LNM was developed in the training set and validated in the test set. Model performance was evaluated by accuracy, balanced accuracy, sensitivity, specificity, positive and negative predictive values (PPV, NPV), and area under the curve (AUC) with 95% confidence intervals (95% CIs). Analyses were conducted in R version 4.0.5, and *p* < 0.05 was considered statistically significant.

## Results

**Figure 1:**
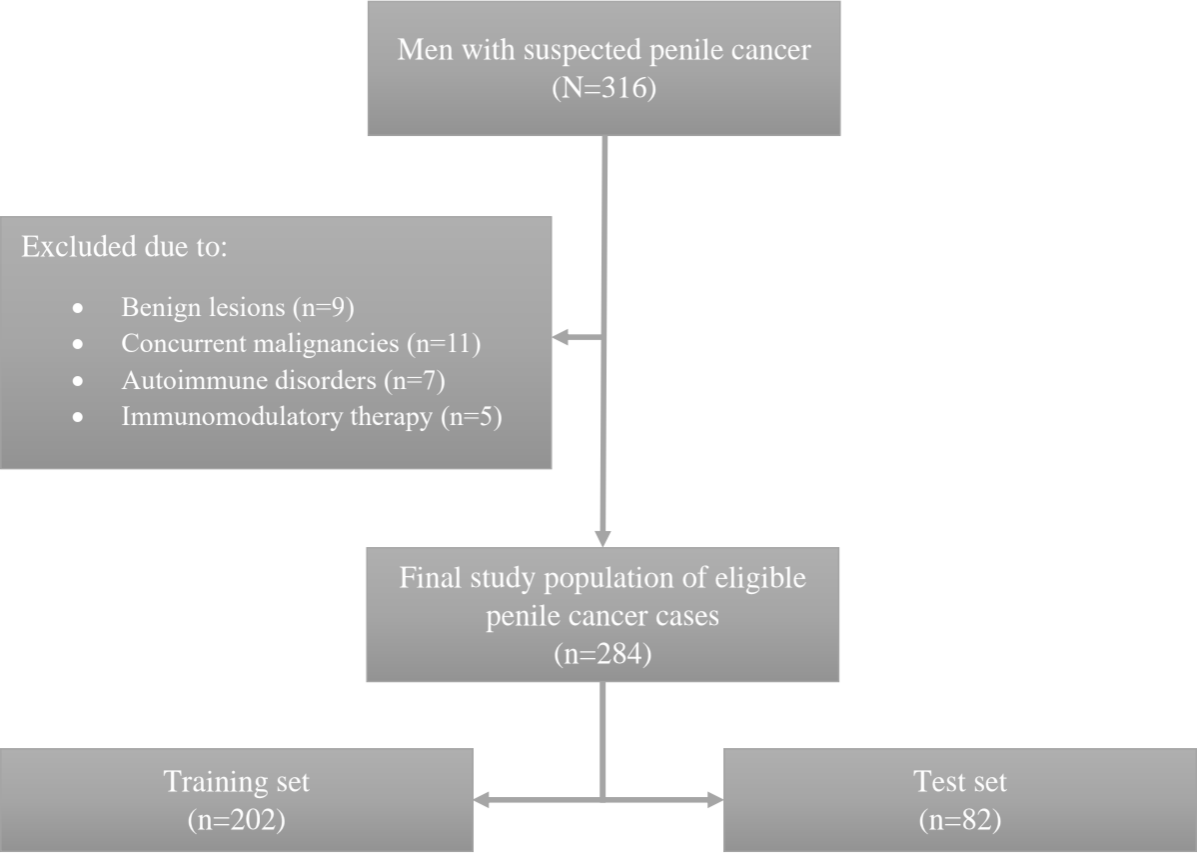
Flowchart outlining the study design with the inclusion and exclusion criteria.

From the initial cohort, 284 PeCa patients and 45 cancer-free controls met the inclusion criteria. Cases were divided into a training set (n=202) and a test set (n=82) (**Figure 1**). Clinicopathological characteristics are presented in **Table 1**. Pathologically confirmed LNM (pN+) were present in 24.1% of the training set and 35.9% of the test set. HPV positivity was observed in 41.6% and 45.6% of cases in the training and test sets, respectively. No statistically significant clinicopathological differences were observed between the two sets.

**Table 1:** Clinicopathological characteristics of men with penile cancer (PeCa), divided into a training and a test set, and a control group of penile cancer-free men.

Characteristic		PeCa training set (n=202)	PeCa test set (n=82)	*p*-value	PeCa-free control group (n=45)
Age at inclusion (years)				0.4^c^	
	Mean (SD)	69.8 (10.6)	68.6 (14.2)		72.7 (8.5)
BMI				0.5^c^	
	Mean (SD)	27.9 (5.3)	28.3 (4.9)		26.7 (3.2)
pT stage, No. (%)				0.3^d^	
	PeIN	26 (12.87)	4 (4.9)		
	pTa	1 (0.49)	0 (0)		
	pT1	66 (32.67)	29 (35.4)		
	pT2	66 (32.67)	27 (32.9)		
	pT3	42 (20.8)	22 (26.8)		
	pT4	1 (0.49)	0 (0)		
pN stage, No. (%)				0.4^d^	
	pN0	142 (75.9)	50 (64.1)		
	pN1	19 (10.2)	14 (17.95)		
	pN2	8 (4.3)	3 (3.85)		
	pN3	18 (9.6)	11 (14.1)		
	pNX^a^	15	4		
Grade, No. (%)				0.08^e^	
	G1	28 (15.9)	5 (6.4)		
	G2	72 (40.9)	43 (55.1)		
	G3	76 (43.2)	30 (38.5)		
	GX^b^	26	4		
HPV-status, No. (%)				0.8^e^	
	HPV+	84 (41.6)	36 (45.6)		
	HPV-	94 (46.5)	43 (54.4)		
	Missing	24 (11.9)	3 (3.3)		

a PeIN, pTa and pT1G1 cases that were not lymph node staged.

b PeIN cases.

c Students T-test.

d Fishers exact test.

e Chi-square test.

Plasma levels of 14 sICs were initially measured in all cases and controls. PD-L1 was excluded due to detectability below the predefined detection threshold (>50% of samples) since 96% of samples did not have detectable PD-L1. Thirteen sICs were thus included for further analyses.

A logistic regression model was developed to predict LNM based on the 13 included sICs. In the training set, the model achieved an accuracy of 77.5% (95% CI: 70.9 – 83.3) and a balanced accuracy of 54.1%. Specificity was high (99.3%), but sensitivity was low (8.9%), with a PPV of 80.0% and a NPV of 77.5% (**Table 2**). When pT-stages and tumor grades were added to the model, accuracy and specificity decreased slightly to 69.6% (95% CI: 60.2 – 78.0) and 85.5% (95% CI: 75.0 – 92.8), respectively, while sensitivity increased to 44.2% (95% CI: 29.1 – 60.1).

**Table 2:** Results of the prediction model for lymph node metastases based on 13 soluble immune checkpoint proteins in penile cancer patients divided into training and test sets.

Dataset		Accuracy % (95% CI)	Balanced ACC %	PPV % (95% CI)	NPV % (95% CI)	TP	FP	TN	FN	Sensitivity % (95% CI)	Specificity % (95% CI)
Training set (n=202)		77.5 (70.9 – 83.3)	54.1	80.0 (31.4 – 97.2)	77.5 (75.9 – 79.1)	4	1	141	41	8.9 (2.5 – 21.2)	99.3 (96.1 – 99.9)
Test set (n=82)		62.2 (50.8 – 72.7)	51.5	38.5 (18.3 – 63.3)	66.7 (62.1 – 71.2)	5	8	46	23	17.9 (6.1 – 36.9)	85.2 (72.9 – 93.4)

ACC, Accuracy; CI, Confidence interval; NPV, Negative predictive value; PPV, Positive predictive value; TP, True positives; FP, False positives; TN, True negatives; FN, False negatives.

**Figure 2:**
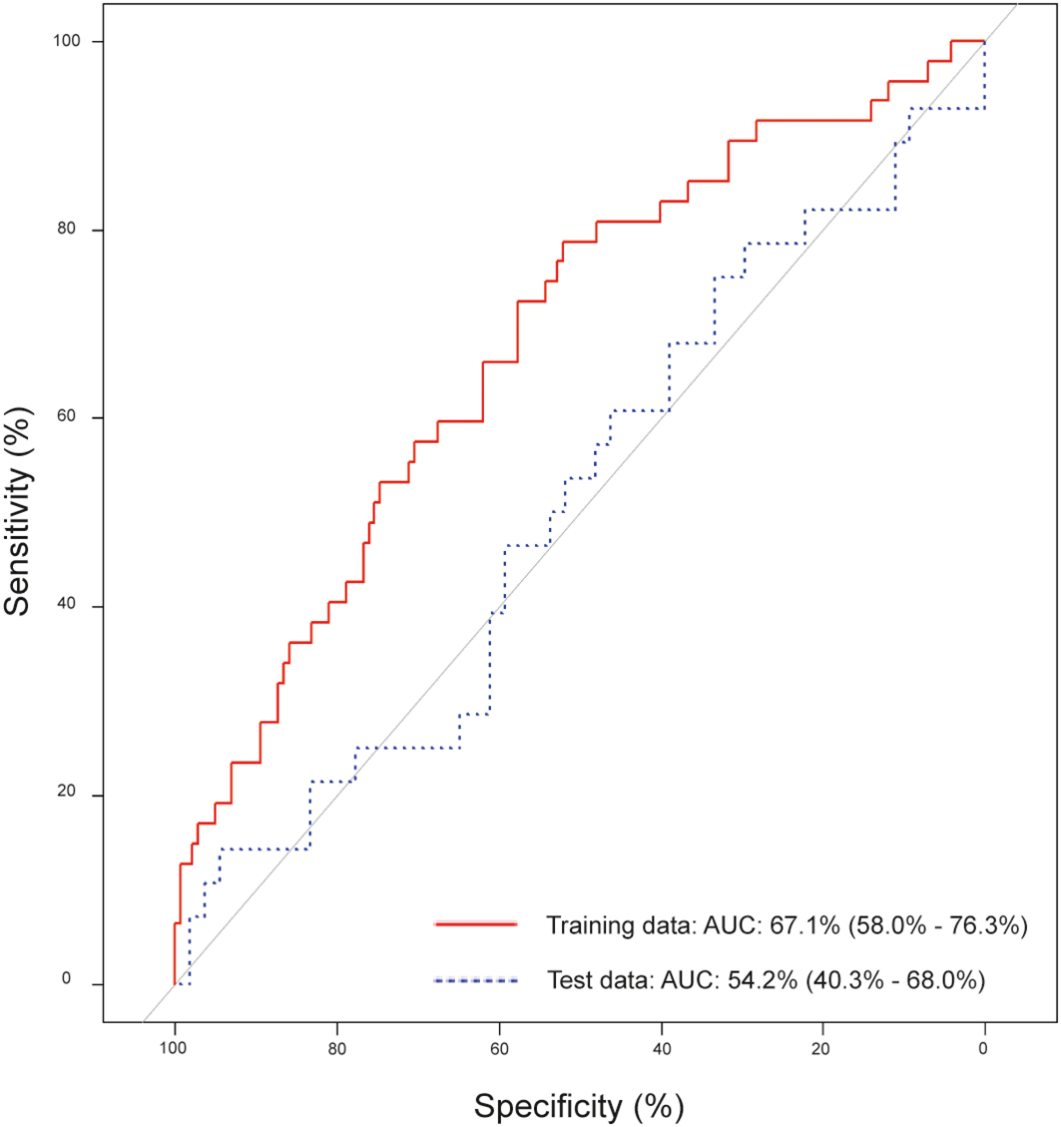
Area under the curve (AUC) for the proposed lymph node metastases prediction model, built using the training data and validated on the test data.

When validated in the test set, model performance declined, yielding an accuracy of 62.2% (95% CI: 50.8 – 72.7), sensitivity of 17.9%, and specificity of 85.2% (**Table 2**). The AUC was 67.1% (95% CI: 58.0 – 76.3) in the training set and 54.2% (95% CI: 40.3 – 68.0) in the test set (**Figure 2**), indicating limited generalizability. Applying the model incorporating pT-stage and tumor grade to the test data resulted in a lower accuracy of 46.9% (95% CI: 32.5 – 61.7) and specificity of 60.7% (95% CI: 40.6 – 78.5), while sensitivity increased to 28.6% (95% CI: 11.3 – 52.2).

To further explore the model´s limited predictive performance, plasma levels of the 13 included sICs were compared between patients with and without LNM within the training set. No statistically significant differences were observed for any sIC (**Table 3a**). Moreover, sIC levels did not vary significantly when the PeCa training set was stratified by pT-stage, tumor grade, or HPV-status (*p* > 0.05, **Supplementary Table 1** and **2**).

**Table 3A:** Concentrations of 13 soluble immune checkpoint proteins (sICs) analyzed in penile cancer (PeCa) patients with (pN+) and without (pN0) lymph node metastases.

sIC	pN0 - Mean concentration (SD)	pN+ - Mean concentration (SD)	*p-*value	Alfa	Significance
BTLA	2539 (14634)	1010 (1398)	0.11	0.044	No
IDO	116 (318)	56 (29)	0.015	0.011	No
LAG-3	229 (1237)	59 (48)	0.052	0.033	No
HVEM	97 (325)	44 (79)	0.036	0.022	No
PD-1	353 (2666)	64 (61)	0.099	0.039	No
PD-L2	1287 (2283)	949 (430)	0.048	0.028	No
TIM-3	1601 (2180)	1373 (854)	0.15	0.05	No
CD80	3227 (13008)	586 (554)	0.0086	0.006	No
CTLA-4 (CD152)	105 (420)	39 (32)	0.033	0.017	No
GITR	239 (788)	131 (87)	0.94	0.05	No
CD27	937 (767)	962 (1339)	0.45	0.013	No
CD28	5224 (43243)	426 (620)	0.91	0.038	No
CD137	190 (526)	135 (86)	0.88	0.025	No

SD = standard deviation, Mean concentration (picogram/milliliter)

### Sub-analyses

To further assess the included sICs potential as systemic biomarkers of tumor-induced immunomodulation, the plasma levels were compared between patients with PeCa and cancer-free controls. Four inhibitory sICs (IDO, TIM-3, CD80, and CTLA-4) were detected at significantly higher mean concentrations in PeCa cases compared to cancer-free controls (**Table 3b**).

**Table 3B:** Concentrations of 13 soluble immune checkpoint proteins (sICs) analyzed in penile cancer (PeCa) patients and penile cancer-free controls.

sIC	PeCa Mean concentration (SD)	Controls Mean concentration (SD)	*p-*value	Alfa	Significance
BTLA	2077 (12295)	845 (924)	0.081	0.039	No
IDO	97 (269)	56 (30)	0.016	0.022	Yes
LAG-3	179 (1039)	52 (40)	0.042	0.033	No
HVEM	81 (276)	31 (19)	0.0058	0.006	No
PD-1	268 (2237)	57 (34)	0.091	0.044	No
PD-L2	1194 (1932)	1004 (369)	0.099	0.05	No
TIM-3	1528 (1884)	1185 (386)	0.009	0.011	Yes
CD80	2469 (10973)	636 (842)	0.010	0.017	Yes
CTLA-4 (CD152)	85 (354)	35 (35)	0.026	0.028	Yes
GITR	212 (669)	116 (79)	0.97	0.038	No
CD27	931 (911)	797 (474)	0.92	0.025	No
CD28	3797 (36286)	382 (661)	0.91	0.013	No
CD137	172 (444)	107 (50)	0.98	0.05	No

SD, standard deviation, Mean concentration (picogram/milliliter)

## Discussion

Penile cancer is a rare but aggressive disease, with prognosis strongly influenced by the presence of LNM. Current clinical tools often fail to predict nodal involvement accurately, highlighting the need for reliable, preferably non-invasive biomarkers. In this study, the predictive potential of 14 sICs for identifying LNM in patients with PeCa was evaluated. A logistic regression model incorporating 13 sICs achieved high specificity in the training set but showed low sensitivity and moderate balanced accuracy. Performance declined further in the independent internal test set, with low AUC values in both cohorts. No significant associations were observed between sIC levels and tumor characteristics, including pT-stage, tumor grade, HPV-status, or LNM. Although plasma sIC profiling remains a non-invasive biomarker approach, its predictive utility for LNM in PeCa appears limited, suggesting that systemic levels of sICs are not primarily driven by tumor aggressiveness.

The predictive model based on 13 detectable sICs demonstrated limited performance already in the training set, with low sensitivity and moderate accuracy despite high specificity. The AUC values (0.67 in the training set and 0.54 in the test set) further indicate poor discriminative capacity, suggesting that systemic levels of sICs alone provide insufficient predictive information for identifying LNM in PeCa. These results imply that sIC profiles may not adequately reflect the local immune dynamics driving metastatic progression.

When pT-stage and tumor grade were added to the model sensitivity increased, whereas accuracy and specificity decreased. This indicates that incorporating established clinicopathological variables slightly improved the model´s ability to identify LNM-positive cases but at the cost of reduced overall accuracy. Thus, even with additional clinical parameters, the model´s predictive capacity remained insufficient for clinical application. The small effect size between LNM-positive and LNM-negative patients likely limited discriminative power, while the pronounced class imbalance (substantially more LMN-negative than LNM-positive cases) may have biased the model toward the majority class, reducing sensitivity for LNM. Although strategies to mitigate class imbalance were explored, these did not substantially improve model performance or sensitivity. Moreover, recent methodological studies indicate that correcting for class imbalance may introduce miscalibration through risk overestimation that is not always correctable, suggesting that such corrections are not necessarily required and may even be detrimental for clinical prediction models (29).

In addition, the training and test sets were analyzed using different multiplex platforms, which may introduce inter-platform variability and influence model validation. However, as no major discrepancy in performance was observed between the training and test sets, this suggests that platform-related differences did not substantially affect overall classification performance, while also providing an initial indication of model robustness across platforms.

Even though categorization of sICs is commonly seen in the literature, and many regression models for predicting outcome are based on categorized sICs, we analyzed them as continuous variables. This decision was based on the absence of biologically or clinically validated cut-offs for the investigated sICs. Arbitrary categorization lacks biological plausibility and leads to loss of information and reduced statistical power (30). Maintaining continuous variables therefore improved the reliability of statistical inference.

Our results differ in some respects from previous studies that have evaluated the potential of using sICs as biomarkers in other solid cancers. In renal cell carcinoma, Wang et al. found elevated sTIM-3 and sLAG-3 levels correlated with advanced disease, sPD-L2 with tumor recurrence, and sTIM-3 and sBTLA with decreased survival (20). Furthermore, Bian et al. found that increased levels of sBTLA, sPD-1, and sPD-L1 were significantly associated with poor prognosis in patients with pancreatic adenocarcinoma and Li et al. demonstrated a correlation between elevated levels of sBTLA and sTIM-3 and disease progression in osteosarcoma patients (18, 19). The observed discrepancies could be explained by methodological variations, such as differences in the assays or detection techniques used to measure sICs. It may also suggest that sICs may reflect tumor aggressiveness in a cancer-specific manner.

In PeCa, immune checkpoint expression has mainly been studied in tumor tissue. PD-L1 expression ranges from 32% to 75% and is linked to adverse features and poor survival (23–26). Davidsson et al. and Ottenhof et al. identified PD-L1 positivity as a predictor of poor survival, with Ottenhof also linking its pattern to LNM (23, 24). These findings were confirmed, showing high PD-L1, TIGIT, and CD155 expression in LNM-positive PeCa, as well as better survival in PD-L1-negative patients (25, 26).

The discrepancy between blood- and tissue-based IC assessments may reflect both biological and methodological differences. Soluble proteins can be affected by systemic inflammation and individual immune variability, and their circulating levels may not reflect the immune interactions within the tumor microenvironment. In addition, the lack of detectable sPD-L1 in this study is most likely due to methodological constraints rather than true biological scarcity. Previous studies using the ProcartaPlex panel have similarly reported low or undetectable sPD-L1 levels (31, 32). In our prior work, sPD-L1 was readily detected in urinary bladder cancer and renal cell carcinoma samples using ELISA, whereas detection was markedly reduced when using the ProcartaPlex panel on the same material (unpublished data). This suggests that differences in assay sensitivity and limit of detection (lower limit of quantification 5.62 pg/mL), rather than limited biological availability, likely explain the low detectability observed here.

Tissue-based assessments, by contrast, allow direct quantification of IC expression in tumor cells and tumor-infiltrating lymphocytes, providing localized insight into immune evasion mechanisms relevant to metastatic progression and greater analytical specificity and sensitivity.

When comparing PeCa patients and cancer-free controls, we found that four inhibitory sICs (IDO, TIM-3, CD80, and CTLA-4) were significantly elevated in the PeCa group. This supports the concept that tumor development is associated with a shift towards an immunosuppressive environment, as inhibitory ICs (such as IDO, TIM-3, CD80, and CTLA-4) are key regulators that limit immune activation. The increased levels observed in patients with PeCa indicate that penile tumors may exploit these immune regulatory pathways to evade immune surveillance. Furthermore, the concurrent elevation of multiple ICs suggests that penile tumors may use multiple pathways to suppress antitumor immune responses and that systemic immunomodulation may occur early in PeCa development as it appeared independent of tumor burden. Although based on a limited sample size, and thus requiring cautious interpretation, our findings are consistent with previous reports across various malignancies, where elevated inhibitory sIC levels are thought to reflect chronic tumor-induced immunosuppression (17–20).

Strengths of this study include its relatively large cohort and inclusion of a cancer-free control group, enhancing comprehensive analyses. Furthermore, we used a standardized multiplex assay for multianalyte profiling, and the study design incorporated both a training set and an independent test set of PeCa patients, which strengthens the reliability of our findings. Limitations include the small number of LNM-positive cases, reducing statistical power.

In conclusion, the evaluated panel of 13 sICs measured in plasma demonstrated limited utility for predicting LNM in PeCa, due to low sensitivity, modest accuracy, and weak discriminative power between LNM-positive and LNM-negative patients. This study nevertheless represents the first systemic evaluation of a broad sIC panel in PeCa. Four inhibitory sICs were significantly elevated in PeCa patients compared to cancer-free controls, suggesting systemic immunosuppression associated with tumor presence, consistent with findings in other malignancies. Future studies assessing larger cohorts and integrating both tissue-based and soluble ICs are warranted to clarify their prognostic significance in PeCa.

## Data Availability

The raw data supporting the conclusions of this article will be made available by the authors, without undue reservation.
