# Supplementary material for: Soluble immune checkpoint proteins as predictive biomarkers for lymph node metastases in penile cancer
Source: Front Immunol. 2026 Feb 2;17:1754254. doi: 10.3389/fimmu.2026.1754254 (PMC12907362; doi:10.3389/fimmu.2026.1754254)
Supplement: Supplementary file 1 [file DataSheet1.pdf]

Supplementary Table 1. Concentrations of 13 soluble immune checkpoint proteins (sICs) analyzed in penile cancer patients in the training set, presented separately for HPV negative (HPV-) and HPV positive (HPV+) cases.

	**HPV-**	**HPV+**			
**sIC**	Mean concentration (SD)	Mean concentration (SD)	*p*-value	Alfa	Significance
BTLA	3216 (17643)	1161 (3807)	0.27	0.042	No
IDO	133 (353)	72 (181)	0.14	0.027	No
LAG-3	325 (1513)	52 (45)	0.08	0.023	No
HVEM	130 (389)	40 (97)	0.03	0.004	No
PD-1	475 (3265)	96 (287)	0.27	0.038	No
PD-L2	1169 (1546)	1294 (2511)	0.69	0.046	No
TIM-3	1806 (2670)	1276 (557)	0.063	0.012	No
CD80	4100 (15412)	1100 (4344)	0.07	0.019	No
CTLA-4 (CD152)	147 (514)	31 (19)	0.031	0.008	No
GITR	204 (380)	237 (948)	0.77	0.05	No
CD27	1063 (1166)	811 (604)	0.068	0.015	No
CD28	7805 (53059)	307 (297)	0.17	0.031	No
CD137	224 (621)	134 (196)	0.18	0.035	No

SD = standard deviation, Mean concentration (picogram/milliliter)

Supplementary Table 2. Concentrations of 13 soluble immune checkpoint proteins (sICs) in penile cancer patients in the training set stratified by pT-stage and tumor grade.

sICs	pT-stage	Mean concentration (SD)	Grade	Mean concentration (SD)
**BTLA**				
	PeIN	845 (566)	G1	896 (444)
	pT1	4507 (21390)	G2	4096 (20488)
	pT2	920 (768)	G3	1020 (1224)
	pT3	893 (720)		
	pT4	979 (NA)		
	pTa	840 (NA)		
**IDO**				
	PeIN	51 (25)	G1	88 (161)
	pT1	161 (451)	G2	139 (424)
	pT2	58 (38)	G3	78 (105)
	pT3	90 (136)		
	pT4	64 (NA)		
	pTa	37 (NA)		
**LAG-3**				
	PeIN	53 (50)	G1	110 (209)
	pT1	328 (1685)	G2	285 (1612)
	pT2	53 (43)	G3	148 (631)
	pT3	228 (844)		
	pT4	78 (NA)		
	pTa	31 (NA)		
**HVEM**				
	PeIN	29 (27)	G1	63 (131)
	pT1	137 (397)	G2	123 (379)

	pT2	41 (70)	G3	65 (242)
	pT3	90 (324)		
	pT4	50 (NA)		
	pTa	23 (NA)		
**PD-1**				
	PeIN	67 (60)	G1	79 (84)
	pT1	644 (3898)	G2	588 (3734)
	pT2	67 (58)	G3	105 (242)
	pT3	130 (316)		
	pT4	44 (NA)		
	pTa	78 (NA)		
**PD-L2**				
	PeIN	1088 (382)	G1	977 (394)
	pT1	1622 (3272)	G2	1508 (3145)
	pT2	865 (309)	G3	1013 (618)
	pT3	1123 (770)		
	pT4	573 (NA)		
	pTa	1013 (NA)		
**TIM-3**				
	PeIN	1339 (648)	G1	1366 (635)
	pT1	1462 (816)	G2	1510 (1362)
	pT2	1563 (2781)	G3	1670 (2725)
	pT3	1721 (1935)		
	pT4	1086 (NA)		
	pTa	922 (NA)		
**CD80**				
	PeIN	753 (1457)	G1	1804 (6170)
	pT1	3950 (12764)	G2	3029 (11634)
	pT2	641 (732)	G3	2771 (13212)
	pT3	4165 (17774)		

	pT4	465 (NA)		
	pTa	742 (NA)		
**CTLA-4 (CD152)**				
	PeIN	30 (21)	G1	92 (231)
	pT1	99 (325)	G2	72 (279)
	pT2	38 (30)	G3	113 (491)
	pT3	174 (658)		
	pT4	53 (NA)		
	pTa	22 (NA)		
**GITR**				
	PeIN	155 (264)	G1	139 (106)
	pT1	317 (1125)	G2	304 (1078)
	pT2	141 (99)	G3	170 (241)
	pT3	195 (314)		
	pT4	228 (NA)		
	pTa	81 (NA)		
**CD27**				
	PeIN	830 (480)	G1	975 (598)
	pT1	1136 (1295)	G2	926 (829)
	pT2	819 (496)	G3	954 (1168)
	pT3	869 (879)		
	pT4	395 (NA)		
	pTa	591 (NA)		
**CD28**				
	PeIN	303 (392)	G1	1867 (6381)
	pT1	9755 (63124)	G2	8436 (60396)
	pT2	424 (599)	G3	1308 (5325)
	pT3	2060 (7101)		
	pT4	485 (NA)		
	pTa	164 (NA)		

**CD137**				
	PeIN	113 (56)	G1	124 (85)
	pT1	261 (765)	G2	242 (731)
	pT2	124 (57)	G3	144 (107)
	pT3	147 (109)		
	pT4	182 (NA)		
	pTa	83 (NA)		

SD = standard deviation, NA = Not applicable (only one patient), Mean concentration (picogram/milliliter)
